# Description of Motor Stereotypies in Adolescents and Adults With Autistic Spectrum Disorder

**DOI:** 10.62641/aep.v53i4.1917

**Published:** 2025-08-05

**Authors:** María Gema Hurtado Ruíz, María Jesús Arranz Calderón, Víctor Pérez Solá, Amaia Hervás Zúñiga

**Affiliations:** ^1^Hospital del Mar Research Institute, Mental Health Group, 08003 Barcelona, Spain; ^2^Brief Hospitalization and Emergency Department, Mental Health Institute, Hospital del Mar, 08003 Barcelona, Spain; ^3^Research Laboratory Unit, Fundació Docència i Recerca Mútua de Terrassa, 08221 Terrassa, Spain; ^4^Department of Health and Life Sciences, Pompeu Fabra University, 08003 Barcelona, Spain; ^5^Biomedical Investigation Center Network of Mental Health (CIBERSAM), 28029 Madrid, Spain; ^6^Fundació Recerca i Docència Mútua de Terrassa, 08221 Terrassa, Spain; ^7^Child and Youth Mental Health Service, Hospital Universitario Mútua de Terrassa, 08221 Terrassa, Spain

**Keywords:** motor stereotypies, repetitive behaviors, autism spectrum disorders, adolescents, adults

## Abstract

**Background:**

Motor stereotypies (MS) are highly prevalent in children with autism spectrum disorders (ASD) and, although they tend to decrease with age, may persist into adulthood. The primary objective of this study was to describe the frequency, severity, number, and types of MS in adolescents and adults with ASD, to retrospectively evaluate their evolution over time, as well as to examine their relationship with sociodemographic and clinical variables.

**Methods:**

A sample of 90 adolescents and adults with ASD were included in a cross-sectional and retrospective study. Rojahn's Stereotypic Behavior Scale (SBS) was used to measure the frequency, severity, and types of MS, while the Achenbach System of Empirically Based Assessment (ASEBA) inventories were utilized to assess psychiatric comorbidity.

**Results:**

MS were observed in 86.5% of cases. The most frequent MS in adolescents and adults with ASD were complex hand and finger movements and pacing (both of which were the most persistent over time) and repetitive body movements (which decreased in periodicity over time). Other, more socially inappropriate MS diminished over time. A significant reduction in the frequency and severity of MS was observed. No correlation was found between age and frequency of MS, and no differences were observed between men and women. Individuals with ASD and intellectual disability (ID) exhibited more types of MS per case and more frequent MS than those without ID, although these differences were not statistically significant. The ASD group with psychopathological comorbidities showed greater frequency and severity of MS, as well as more types of MS per case.

**Conclusions:**

MS decreased in frequency and severity over time but persisted in ASD, particularly those that are more specific to ASD. The most socially inappropriate MS tended to disappear. The presence of MS in adolescents and adults with ASD was not influenced by age or sex. Adolescents and adults with ASD and ID or psychopathological comorbidities exhibited a greater variety of stereotypies, with the psychopathological comorbidities group showing higher frequency and severity of MS. Understanding the characteristics of MS could aid in predicting their progression, designing more targeted treatments (if needed), and identifying phenotypic subgroups to facilitate the discovery of associated risk genes.

## Introduction

Autism spectrum disorders (ASD) are a group of neurodevelopmental disorders characterized by persistent deficits in reciprocal social communication and interaction, along with restrictive and repetitive patterns of behavior, interests, or activities (i.e., restrictive and repetitive behaviors (RRBs)). These features manifest in early development and cause clinically significant impairment in various domains of daily functioning [1].

Classically, RRBs have been categorized into low-level behaviors—sensorimotor repetitive behaviors, such as motor stereotypies (MS), object manipulation, and repetitive self-injurious behaviors—and high-level behaviors, such as object attachment, insistence on sameness, routines, repetitive language, and circumscribed interests [2, 3]. These two categories are associated with distinct clinical, neuroanatomical, and genetic features, leading several authors to propose that they should be studied separately [4, 5, 6].

MS are present from early childhood [7, 8] and occur frequently in individuals with ASD, with a median prevalence of 51.8% (range, 21.9%–97.5%) according to the meta-analysis by Melo *et al*. [9] in 2019. These authors defined MS as “a hyperkinetic movement disorder, characterized by involuntary, patterned, coordinated, purposeless, ritualistic movements, postures, or utterances, repeated continuously for a period of time in the same form and on multiple occasions, and which are distractible in the majority of cases”. However, other authors suggest avoiding the qualifiers “voluntary”, “suppressible”, and “distractible” until more evidence is available [3, 9]. MS are further characterized by the absence of a prior sense of urgency before their performance [10] and their predictability in form, amplitude, and location [11].

Some definitions of MS include the absence of a clear purpose in performing these behaviors [9], however, some authors have observed that MS in ASD may serve various functions, such as arousal seeking, sensory processing and seeking, reducing anxiety, improving emotional regulation, enhancing attention and concentration, gaining tangible objects, attention seeking, or escape [10, 12, 13, 14, 15]. These motivational factors favor the development and maintenance of MS, with their influence depending on the developmental stage [4], type of repetitive behavior [16], context [15, 17], and underlying pathology [4].

MS are not specific to ASD and are observed in a wide range of conditions, including congenital syndromes, neurological diseases, psychiatric disorders, language disorders, sensory deficits, and intellectual disability (ID). They can also be induced by substances or sensory deprivation and are present in neurotypical children during the first 4 years of life, with a tendency to decrease after the age of 2 years [4]. In ASD, children under 2 years of age already exhibit higher levels of MS [18, 19, 20]. These behaviors persist at similar levels beyond this period, encompassing a wide range of behaviors and locations. They are often complex, atypical, or unusual, occurring with greater severity and frequency than in other disorders, and they interfere with the individual’s functioning [9, 13, 21, 22, 23, 24, 25]. Certain MS, such as hand/finger movements and gait stereotypies, appear almost exclusively in children with ASD [24, 25]. By contrast, head and trunk stereotypies are more commonly associated with a non-verbal intellectual coefficient (IQ) below 80 [24].

The presence of MS in ASD may be influenced by clinical and sociodemographic factors, with a higher prevalence observed in children. This prevalence tends to decrease with age [9, 26], possibly because of social stigma or the replacement of MS with other, more adaptive RRBs [27]. MS are also more prevalent in individuals with ID [9]. No influence of sex has been identified with respect to the presence of MS [9]. MS has been associated with reduced adaptive behavior [28, 29], poorer motor skills, greater ASD severity [9, 30], emotional dysregulation [31], anxiety [32, 33], and impaired sensory reactivity [34]. However, studies on the relationship between MS and impaired executive functions have yielded contradictory results [35].

MS represent the most basic behaviors within sensorimotor repetitive behaviors, serving as a phenotype closely linked to the genetic alterations that may underlie them. They are easily observable and measurable in clinical settings, with an identifiable neural network and the ability to be reproduced in animal models [36]. Improved recognition, description, and classification of stereotypies is important for planning therapeutic interventions and advancing research on neuroanatomical locations and biological mechanisms [9]. Additionally, studying MS specifically helps address the challenges of classifying RRB [37]. MS have been extensively studied in children with ASD, in whom they are more prevalent. Although MS decrease with age, they may persist into adolescence and adulthood [4, 26].

This study was performed to examine MS in a sample of adolescents and adults with ASD in terms of frequency, number, severity, duration, and typology. Changes in MS over time were examined, and differences in the presentation of MS based on age, sex, ID, and the presence of psychopathological comorbidities were analyzed.

## Methods

### Study Design

A cross-sectional observational study was conducted to describe the number, typology, frequency, and severity of MS in adolescents and adults with ASD. Additionally, a retrospective study was carried out to examine the duration of MS and changes over time.

The diagnosis of ASD was established by clinical experts using assessments based on the criteria outlined in the Diagnostic and Statistical Manual of Mental Disorders, Fourth Edition, Text Revision or the Diagnostic and Statistical Manual of Mental Disorders, Fifth Edition. The diagnosis was confirmed in most cases with the Autism Diagnostic Interview-Revised [38] and the Autism Diagnostic Observation Schedule [39].

Recruitment was conducted between 2021 and 2022 through the Child and Youth Mental Health Service at Hospital Universitario Mútua de Terrassa, Global institute of neurodevelopment integrated care (IGAIN), two autism associations in Barcelona, and a special education school specializing in autism in Barcelona. The inclusion criteria for patients with ASD were being over 14 years of age, meeting the criteria for ASD according to the *Diagnostic and Statistical Manual of Mental Disorders, Fifth Edition* as confirmed by expert clinicians, and having any intellectual level. The exclusion criteria were a history of severe head injury and any type of family conflict that hindered collaboration in the study.

### Measuring Instruments

The questionnaires were completed online using the Jotform platform https://www.jotform.com.

#### Achenbach System of Empirically Based Assessment (ASEBA) Inventories 

Age-appropriate versions of the ASEBA inventories were administered to assess psychopathological comorbidities. The Child Behavior Checklist for Ages 4–18 (Spanish version) [40] is a scale designed for parents of individuals aged 4 to 18 years and includes 113 items. The Adult Behavior Checklist for Ages 18–59 (Spanish version) [41] is a scale intended for relatives of individuals aged 18 to 59 years and consists of 126 items. The Adult Self-Report for Ages 18–59 (Spanish version) [41] is a self-administered scale containing 124 items.

Each scale includes cut-off points for clinically significant behavior: scores above 70 (T-score >70) on the eight subscales and scores above 63 (T-score >63) on the three higher dimensions (internalization, externalization, and total). For the Adult Behavior Checklist and Adult Self-Report scales, Spanish norm-referenced scales are not yet available, so these assessments must be corrected using American norm-referenced scales.

#### Stereotyped Behavior Scale (SBS)

The SBS [42] was used to assess MS. It is a scale designed for adolescents and adults with ID and other neurodevelopmental disorders, consisting of 24 items, each describing a type of stereotyped behavior rated on both a frequency scale and a severity scale. A cut-off score above 14–17 (PT = 50) on the frequency scale is considered unusually high, though its clinical relevance remains uncertain.

We developed a modified version of the SBS, referred to as the SBS-MS, which calculates scores based on the sum of motor stereotypy items (items 16, 18–23, 25, 28, 30, 31, 33, 34, and 36–40) occurring in the present, or in the past (more than 1 year ago). Consequently, we obtained 4 scores for the SBS-MS scale: Frequency of SBS-MS currently (SBS-MS-F-C), Severity of SBS-MS currently (SBS-MS-S-C), Frequency of SBS-MS in the past (SBS-MS-F-P) and Severity of SBS-MS in the past (SBS-MS-S-P).

Using the SBS-MS-F-C scale, we studied the prevalence of MS and daily MS (defined as a score indicating at least monthly or daily periodicity on at least one SBS-MS-F-C item per individual). Additional analyses included the current and past periodicity of each MS, the maximum periodicity of MS in each individual, the number of MS types per case, and the number of daily MS types per case [43].

For each SBS-MS-F item, the duration of each MS and the total MS duration for each individual were calculated based on the difference between the age at onset and the age at cessation of the MS (using a cut-off score of at least 1 for onset and 0 for cessation). The percentage of years lived with MS was assessed as the relative duration of MS in relation to the individual’s age. For each type of MS and for total MS, a variable was created to indicate whether MS had become extinct in each individual.

### Statistical Analysis

For the cross-sectional study, a descriptive analysis was performed, calculating frequencies, percentages, means, and standard deviations for normally distributed variables, or medians and interquartile ranges (IQRs) for non-normally distributed variables. The Shapiro-Wilk test was used to verify the normality of the variables. Subgroup comparisons were conducted using the chi-square test for categorical variables and either the parametric Student’s *t*-test or the non-parametric Mann–Whitney U test for continuous variables, as appropriate.

For the retrospective study on the evolution of MS over time, the non-parametric Wilcoxon test for paired data was used. Kaplan–Meier curves were generated to analyze the persistence of MS throughout life, both for individual types of MS and for MS overall.

Statistical analyses were conducted using IBM SPSS Statistics version 22 (IBM, Armonk, NY, USA) and R statistical software version 3.6.3 (R foundation for statistical computing, Vienna, Austria).

## Results

### Participants

Ninety individuals with ASD (69 male, 21 female) aged 14 to 43 years (mean age, 20.6 ± 6.5 years) were included in the study. Of the 90 participants, 52 were adults. In total, 35.6% of the participants had ID and 66.7% were fluent in speech. A medical history was reported in 25.5% of the participants, and 67.7% had a psychiatric history, with attention-deficit/hyperactivity disorder being the most prevalent (43.3%). Clinically significant psychopathology, as measured by the ASEBA inventories, was present in 47.1% of participants, with 47.1% exhibiting clinically significant internalizing behaviors and 29.9% clinically significant externalizing behaviors. Psychopharmacological treatment was used by 78.8% of participants, with 36.7% receiving antipsychotic treatment and 34.4% receiving treatment for attention-deficit/hyperactivity disorder. Additionally, 93.3% had undergone psychotherapy during childhood (Table 1). Data for ASEBA inventory variables were missing in three patients.

**Table 1.** **Summary of participants characteristics**.

Sociodemographic and clinical characteristics			Total sample
			N = 90
Sociodemographic characteristics:			
	Age, Mean (SD)		20.6 (6.5)
	Age ≥18 years old, N (%) Yes/No		52 (57.8)/38 (42.2)
	Sex, N (%) Male/Female		69 (76.7)/21 (23.3)
	Ethnicity, N (%) Caucasian/Latin/Others		87 (97.8)/2 (2.2)/0
	Social class, N (%) Upper middle/Lower middle/Working/Lower class		28 (31.1)/36 (40)/26 (28.9)/0
Clinical characteristics: N (%)			
	ID (IQ ≤70) Yes/No		32 (35.6)/58 (64.4)
	Verbal level Fluent speech/Simple sentences/No verbal o single words/unknown		60(66.7)/17 (18.8)/4 (4.4)/9 (10)
	Medical history		23 (25.5)
	Psychiatric history		61 (67.7)
		ADHD	39 (43.3)
		Anxiety disorder	14 (15.5)
		Depressive disorder	11 (12.2)
	Psychotropic drugs intake		71 (78.8)
		ADHD treatment	31 (34.4)
		Atypical antipsychotics	33 (36.7)
		Antidepressants	13 (14.4)
		Antiepileptics and mood stabilizers	7 (7.8)
	Completion of psychotherapy in childhood		84 (93.3)

SD, Standard deviation; ID, Intellectual disability; IQ, intellectual coefficient; ADHD, Attention deficit/hyperactive disorder.

### Cross-sectional Study

#### Quantitative Description of MS

Table 2 presents the frequency and severity scores of the SBS-MS scale at present. MS were observed in 86.7% of cases at least monthly and in 55.6% of cases on a daily basis (every day and every hour). Additionally, four or more MS per individual were observed in 62.2% of cases at least monthly and in 25.6% of cases daily (Table 2).

**Table 2.** **Quantitative description of motor stereotypies**.

MS variables		Total sample
		N = 90
SBS-MS-F-C		
	Median (IQR)	11 (15)
SBS-MS-S-C		
	Median (IQR)	7 (8)
Prevalence of MS		
	N (%)	78 (86.7)
Maximum periodicity of MS per case		
	N (%) Never/Every month/Every week/Every day/Every hour	12 (13.3)/7 (7.8)/21 (23.3)/41 (45.6)/9 (10)
Number of MS types per case		
	N (%) 0/1–3/4–7/>7	12 (13.3)/22 (24.4)/38 (42.2)/18 (20)
Number of daily MS types per case		
	N (%) 0/1–3/4–7/>7	39 (43.3)/28 (31.1)/18 (20)/5 (5.6)

MS, Motor stereotypies; SBS-MS, Stereotyped Behavior Scale modified version; SBS-MS-F-C, Current frequency score of SBS-MS; SBS-MS-S-C, Current severity score of SBS-MS; IQR, Interquartile range.

#### Relationship of MS With Age

There was no correlation between age and the frequency or severity of MS in adolescents and adults with ASD (Spearman coefficient = 0.006, *p* = 0.957 and Spearman coefficient = 0.009, *p* = 0.929, respectively).

#### Sex-related Differences in MS

The comparison groups did not differ with respect to age, percentage of adults in the sample, presence of psychiatric history, need for support, language fluency, presence of ID, completion of psychotherapy in childhood, treatment intake, or presence of clinically significant psychopathology (Table 3).

**Table 3.** **Men and women ASD groups characteristics**.

Sociodemographic and clinical characteristics		Female	Male	χ^2^/U	*p*
		N = 21	N = 69		
Age, Mean (SD)		18 (14)	19 (8)	763	0.712
Age ≥18 years old, N (%)		11 (52.4)	41 (59.4)	0.327	0.567
ID (IQ ≤70), N (%)		8 (38.1)	24 (34.8)	0.077	0.781
Fluent speech, N (%)		12 (57.1)	48 (69.6)	1.118	0.290
Psychiatric history, N (%)					
	ADHD	7 (33.3)	32 (46.4)	1.115	0.291
	Depression disorder	5 (23.8)	6 (8.7)	3.428	0.120
	Anxiety disorder	5 (23.8)	9 (13)	1.421	0.302
Psychotropic drugs intake, N (%)		16 (76.2)	44 (64.7)	0.963	0.326
Completion of psychotherapy in childhood, N (%)		18 (85.7)	66 (97.1)	3.894	0.083
ASEBA inventory (clinically significant), N (%)		N = 20	N = 67		*p*
	Internalization	9 (45)	32 (47.8)	0.047	0.828
	Externalization	5 (25)	21 (31.3)	0.296	0.587
	Total	10 (50)	31 (46.3)	0.086	0.769

ID, Intellectual disability; ADHD, Attention deficit/hyperactive disorder; ASEBA, Achenbach System Evidence Based Assessment; χ^2^, Chi-Square test; U, U-Mann Whitney test; SD, Statistical deviation.

There were no statistically significant differences between men and women regarding the prevalence, frequency, severity, or types of MS per case in adolescents and adults with ASD (Table 4).

**Table 4.** **Motor stereotypies as a function of sex, intellectual disability and psychopathological comorbidity**.

		Sex			ID			Psychopathological comorbidity		
		Women	Men	χ^2^/U	No	Yes	χ^2^/U	No	Yes	χ^2^/U
		N = 21	N = 69	*p*	N = 58	N = 32	*p*	N = 46	N = 41	*p*
SBS-MS-F-C				568			722			672
	Median (IQR)	15 (8.25)	10 (13)	0.135	9 (13)	14 (17)	0.082	8.5 (11.25)	14 (12.50)	**0.021**
SBS-MS-S-C				637			711.5			512.5
	Median (IQR)	8 (4)	6 (10)	0.402	6 (9)	9 (6)	0.067	6 (7)	10 (11)	**0.001**
Prevalence of MS				0.268			0.723			0.974
	N (%)	18 (90)	59 (85.5)	1.000	48 (84.2)	29 (90.6)	0.525	38 (82.6)	36 (90)	0.324
Number of MS types per case				512			662.5			660
	Median (IQR)	6 (4)	4 (5)	0.165	4 (5)	5.5 (5)	**0.040**	4 (5)	5 (5)	**0.022**
Number of daily MS types per case				650			590			601
	Median (IQR)	2 (5)	1 (3)	0.680	0 (3)	3 (7)	**0.004**	0 (3)	3 (5)	**0.004**

MS, Motor stereotypies; SBS-MS, Stereotyped Behavior Scale modified version; SBS-MS-F-C, Current frequency score of SBS-MS; SBS-MS-S-C, Current severity score of SBS-MS; ID, Intellectual disability; χ^2^, Chi-Square test; U, U-Mann Whitney test; IQR, Interquartile range. In bold *p* < 0.05.

#### Differences in MS in Individuals With ASD With/Without ID

The comparison groups did not differ with respect to age, sex, percentage of adults in the sample, or presence of psychiatric history. Statistically significant differences were found in language fluency (*p* = 0.001), treatment intake (*p* = 0.037), and the presence of externalizing behaviors, which were higher in the ASD with ID group (*p* = 0.021) (Table 5).

**Table 5.** **Characteristics of ASD groups in relation to intellectual disability**.

Sociodemographic and clinical characteristics		Without ID	With ID	χ^2^/U	*p*
		N = 58	N = 32		
Age, Median (IQR)		20 (9)	18 (6)	763	0.163
Age ≥18 years old, N (%)		35 (60.3)	17 (53.1)	0.441	0.507
Sex (Female), N (%)		13 (22.4)	8 (25)	0.077	0.781
Fluent speech, N (%)		10 (17.2)	48 (82.8)	19.009	**0.001**
Psychiatric history, N (%)					
	ADHD	29 (50)	10 (31.3)	2.952	0.086
	Depression disorder	8 (13.8)	3 (9.4)	0.375	0.540
	Anxiety disorder	9 (15.5)	5 (15.6)	0.001	1.000
Psychotropic drugs intake, N (%)		34 (59.6)	26 (81.3)	4.353	**0.037**
Completion of psychotherapy in childhood, N (%)		55 (96.5)	29 (90.6)	1.330	0.346
ASEBA inventory (clinically significant), N (%)		N = 56	N = 31		*p*
	Internalization	27 (48.2)	14 (45.2)	0.750	0.785
	Externalization	12 (21.4)	14 (45.2)	5.364	**0.021**
	Total	24 (42.9)	17 (54.8)	1.150	0.284

ID, Intellectual disability; ADHD, Attention deficit/hyperactive disorder; ASEBA, Achenbach System Evidence Based Assessment; χ^2^, Chi-Square test; U, U-Mann Whitney test; QR, Interquartile range. In bold *p* < 0.05.

The ASD with ID group showed a greater number of MS types per case compared with the ASD without ID group. While the ASD without ID group had no daily MS (median = 0), the ASD with ID group had a median of three different daily MS per case. Although both groups differed in SBS-MS scores for frequency and severity, the differences were only marginally significant. There were no statistically significant differences in the prevalence of MS between the groups (Table 4).

#### Differences in MS in Individuals With ASD With/Without Psychopathological Comorbidities 

The comparison groups did not differ with respect to age, sex, percentage of adults, presence of psychiatric history, language fluency, presence of ID, or completion of psychotherapy in childhood (Table 6).

**Table 6.** **Characteristics of ASD groups in relation to psychopathological comorbidity**.

Sociodemographic and clinical characteristics		With PSYCH	Without PSYCH	χ^2^/U	*p*
		N = 41	N = 46		
Age, Median (IQR)		19 (9)	18.5 (9)	904	0.739
Sex (Female), N (%)		10 (24.4)	10 (21.7)	0.086	0.769
Age ≥18 years old, N (%)		26 (63.4)	25 (54.3)	0.735	0.391
ID (IQ ≤70), N (%)		17 (41.5)	14 (30.4)	1.150	0.284
Fluent speech, N (%)		26 (63.4)	32 (69.6)	0.369	0.544
Psychiatric history, N (%)					
	ADHD	18 (43.9)	20 (43.5)	0.002	0.968
	Depression disorder	10 (24.4)	1 (2.2)	9.687	**0.002**
	Anxiety disorder	11 (26.8)	3 (6.5)	6.621	**0.010**
Psychotropic drugs intake, N (%)		34 (85)	24 (52.2)	10.499	**0.001**
Completion of psychotherapy in childhood, N (%)		37 (92.5)	44 (95.7)	0.388	0.533
ASEBA inventory (clinically significant), N (%)		N = 41	N = 46		*p*
	Internalization	33 (80.5)	8 (17.4)	34.636	**0.001**
	Externalization	25 (61)	1 (2.2)	35.772	**0.001**

ID, Intellectual disability; ADHD, Attention deficit/hyperactive disorder; ASEBA, Achenbach System Evidence Based Assessment; PSYCH, psychopathological comorbidity (clinically significant total dimension of ASEBA inventory). χ^2^, Chi-Square test; U, U-Mann Whitney test; IQR, Interquartile range. In bold *p* < 0.05.

There were no statistically significant differences in the prevalence of MS between the groups with and without psychopathological comorbidities. However, statistically significant differences were observed between the groups in terms of frequency, severity, types of MS per case, and types of daily MS per case, with higher scores in the group with psychopathological comorbidities (Table 4).

### Retrospective Study

#### Types of MS: Periodicity Now and in the Past

**Fig. 1.**
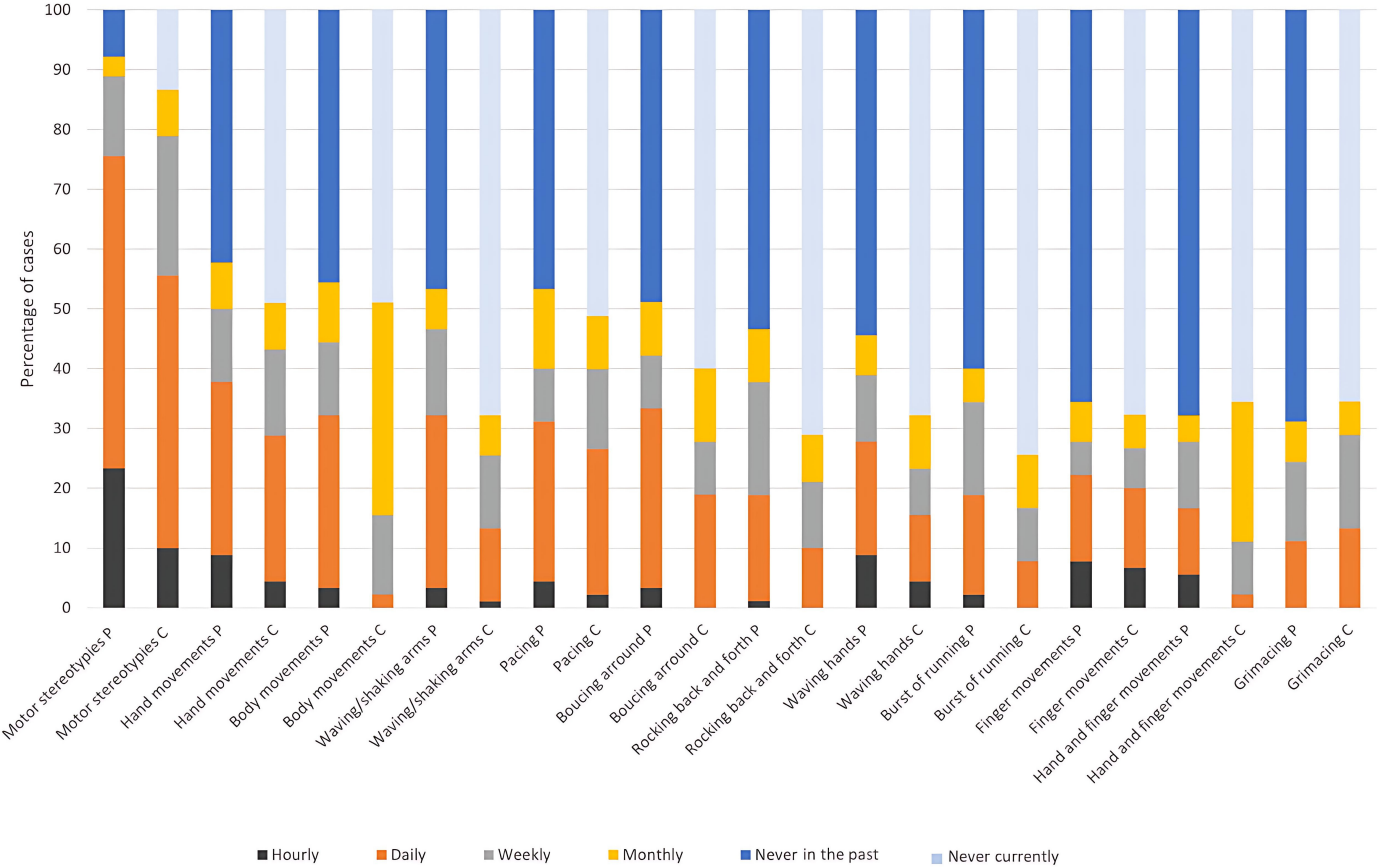
**Past and current periodicity of motor stereotypies (MS)**. The first two columns show the frequency of the past and current maximum periodicity of MS in each individual. The following columns show a comparison between past and present periodicity of different types of MS, ordered by relevance. In general, a reduction in the prevalence and periodicity of each MS is observed, but the percentage of extinction does not exceed 20%.

Fig. 1 illustrates the periodicity of the total and most frequent types of MS, both currently and in the past.

Currently, the most prevalent MS observed at least monthly and on a daily basis were hand movements and pacing. By contrast, repetitive body movements, while common on a monthly basis, were less frequently observed daily. Among all types, hand-related movements were the most frequent on an hourly basis.

Overall, the periodicity of all MS types had decreased compared with the past. However, some MS, such as complex hand and finger movements, remained stable. Conversely, there was a notable decline in arm and hand waving, bouncing, rocking back and forth, and bursts of running.

#### Duration of MS Across the Lifespan

The median duration of MS with a periodicity of at least monthly was 14 years (IQR = 9), with a minimum duration of 1 year and a maximum of 40 years. This corresponds to a median percentage of years lived with MS of 82.35% (IQR = 47%), ranging from a minimum of 5% to a maximum of 96%.

#### Permanence of Each Type of MS

**Fig. 2.**
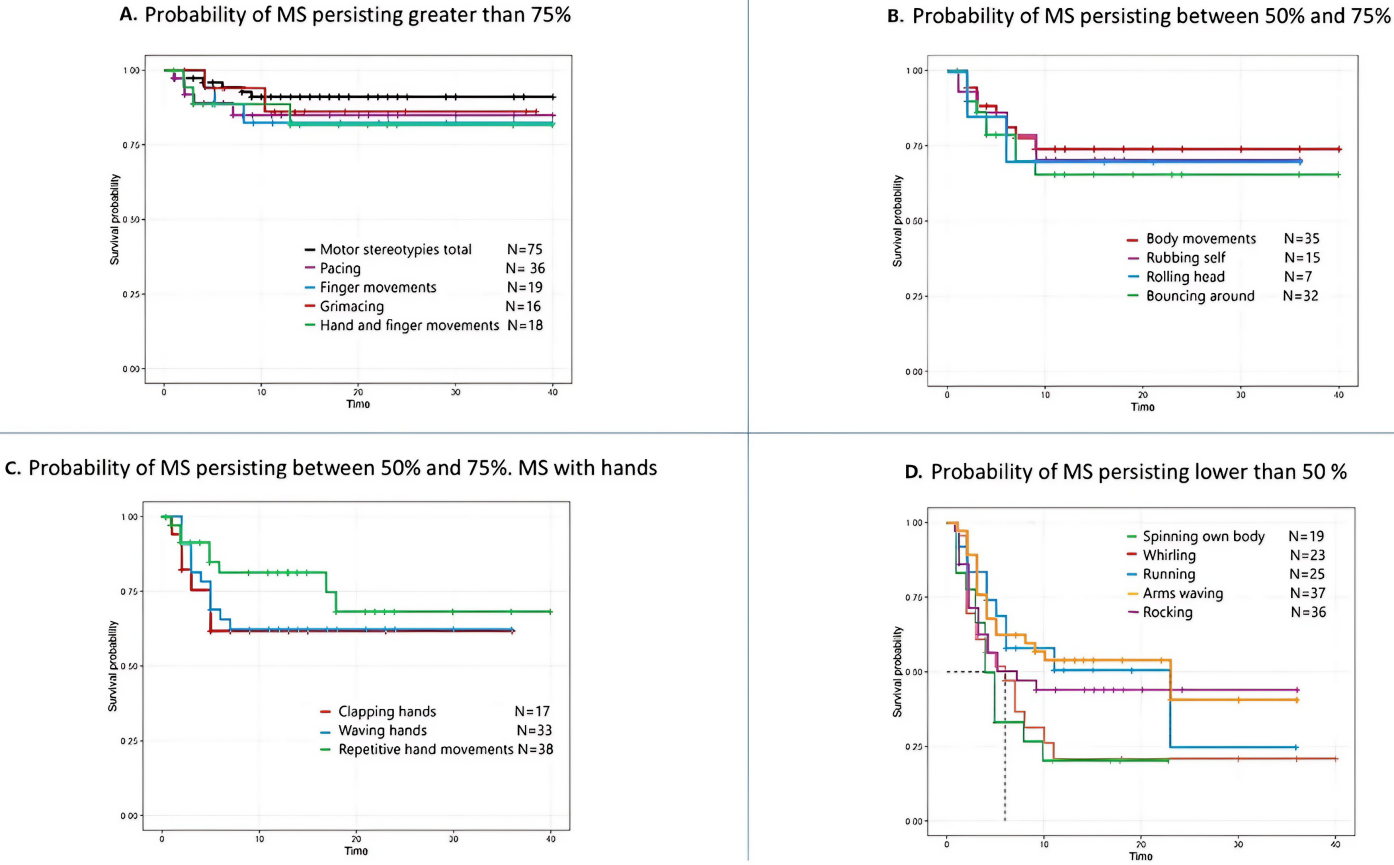
**Probability of motor stereotypies (MS) persisting over time**. Graphs (A–D) show the evolution of MS over time and the likelihood of persisting into adolescence and adulthood. Graph (A) shows those MS that have a probability greater than 75% (the probability of overall MS is included), graphs (B) and (C) show the MS with a probability between 50 and 75% (graph C includes hand movements) and graph (D) shows those MS that have a probability less than 50% of persisting over time.

The Kaplan–Meier survival curves indicated that the probability of MS persisting in total after 10 years from onset was greater than 75%. This was also true for certain types of MS, such as pacing, grimacing, complex hand and finger movements, finger movements, and repetitive body movements. By contrast, stereotypies such as rocking, whirling or turning around on spot, or spinning own body had an approximate 50% probability of persisting at 5 years from onset. Similarly, behaviors such as arm waving or bursts of running around had an approximate 50% probability of persisting after 10 years from onset (Fig. 2).

#### Changes in Presence of MS Over Time

A significant reduction in the SBS-MS-F scale score was observed in the present compared with the past (Wilcoxon test: Z = –6.329, *p* = 0.001). A similar trend was found for the SBS-MS-S scale score (Wilcoxon test: Z = –6.508, *p* = 0.001) (Table 7).

**Table 7.** **Changes in presence of MS over time**.

		N	Median (IQR)	Min	Max	Positive rank sum test	Z	Sig. (bilateral)
SBS-MS-F								
	SBS-MS-F-P	90	16.5 (16)	0	46	2264	–6.329	<0.001
	SBS-MS-F-C	90	11 (15)	0	46			
SBS-MS-S								
	SBS-MS-S-P	90	11 (9.5)	0	31	2066	–6.508	<0.001
	SBS-MS-S-C	90	7 (8)	0	31			

MS, Motor stereotypies; SBS-MS, Stereotyped Behavior Scale modified version; SBS-MS-F-C, Current frequency score of SBS-MS; SBS-MS-S-C, Current severity score of SBS-MS; SBS-MS-F-P, Past frequency score of SBS-MS; SBS-MS-S-P, Past severity score of SBS-MS; IQR, Interquartile range.

## Discussion

This study revealed a high percentage of MS in adolescents and adults with ASD, with several types of MS per case being common. The most prevalent MS were those related to hand movements and pacing. The duration varied depending on the type of MS, and there was a high likelihood of lifelong MS. There was no significant influence of age, sex, or ID (with a marginal difference in this case) on the frequency and severity of MS. Differences were observed in the types of MS per case between groups with and without ID and between those with and without psychopathological comorbidities. MS were more frequent and severe in the group with psychopathological comorbidities than in the group without. A reduction in the frequency and severity of MS over time was also noted.

The presence of MS in our study was close to 90%. This finding aligns with the prevalence of MS in ASD reported in the meta-analysis by Melo *et al*. [9] in 2019. It also underscores that a significant percentage of cases in our sample exhibited four or more different types of MS (25.8% daily and 61.8% at least monthly). The presence of more types of MS per case was associated with the presence of ID (this observation aligns with the results reported by Melo *et al*., 2023 [44]) and psychopathological comorbidities.

The most prevalent and frequent types of MS were hand movements and pacing. Repetitive hand movements and gait-related MS have been described by other authors as the most specific to ASD [24, 25]. Furthermore, these specific MS tended to persist over time, in contrast to repetitive body MS (non-ASD-specific MS [25]), which were also prevalent both currently and in the past but shifted from daily to monthly periodicity over time. Whether these specific types of MS are more strongly associated with particular neuroanatomical or genetic alterations, as observed in some congenital syndromes with ASD (e.g., hand flapping in Angelman syndrome, chewing in Phelan-McDermid syndrome, or hand movements in Rett syndrome), could be explored [45, 46]. Such investigations could help determine whether these behaviors could be considered phenotypic features of ASD.

Compared to MS in the past, we observed a reduction in MS likely related to seeking enhanced sensation [17], including arm and hand waving, bouncing, rocking back and forth, and bursts of running. These behaviors could also be considered highly socially inappropriate. This observation suggests that individuals with ASD, as they develop greater cognitive and motor sophistication with age, gain insight or receive therapies aimed at masking these MS, replace them with more adaptive repetitive or restrictive behaviors [30, 47] and avoid MS in public due to social stigma [27]. Given that our sample predominantly consisted of men, we could infer that not only women but also men exhibit a tendency to mask MS. Additionally, it is possible that the context or motivation triggering and sustaining these MS changes with age [4, 12, 13]. Further research is needed to explore the context in which MS occur in adolescent and adult populations with ASD and the roles they play, as first-person accounts suggest that MS may serve an adaptive function [10, 14].

In the retrospective analysis, we observed a reduction in the frequency and severity scores of the SBS-MS scale compared MS in the past, consistent with the reduction in MS with age described by Melo *et al*., 2019 [9] and Melo *et al*., 2023 [44]. However, although MS decreased and some types disappeared, they persisted in most of our adolescent and adult participants. Notably, the percentage of years lived with MS exceeded 80% on average. In the Kaplan–Meier survival curves, we observed that the disappearance of MS, when it occurred, typically happened within the first 10 years after onset. The persistence of MS beyond this 10-year period may explain the lack of correlation between age and the frequency or severity of MS in our adolescent and adult sample. In ASD, it has been reported that the relationship between age and MS is more evident in early childhood and diminishes over time [48, 49]. In 2009, Esbensen *et al*. [26] found that in ASD without ID, the prevalence of MS differed between children and adolescents but not between adolescents and adults.

The maintenance of MS over time could be attributed to the fundamental involvement of the cortical-basal ganglia-thalamus (CGBT) circuit and other regions such as the reward circuit, hippocampus, and cerebellum [5, 50, 51, 52]. This suggests an altered balance between habitual and goal-directed action control at the root of MS, affecting the motor/premotor cortex and prefrontal cortex, respectively [53]. At the molecular level, potential dysfunctions of the dopaminergic, GABAergic, cholinergic, and glutamatergic systems within the CGBT circuit have been reported [7, 54]. The genetic contribution to the manifestation of MS in ASD is well-supported by evidence derived from animal models, the observation of stereotypies across a spectrum of genetic disorders, and familial aggregation studies [4].

Furthermore, MS may be perpetuated by the reinforcing effect of sensory stimulation resulting from repetitive movement, which could enhance the sensitisation of low-level brain structures that control motor behaviour in the absence of normal inhibitory regulation by higher nervous functions [16, 55, 56, 57, 58]. Repetitive movements, whether direct or through sensory feedback, could regulate asynchronous sensorimotor rhythms in ASD, improving sensory processing and attention [10].

It has been hypothesized that MS are replaced by alternative behaviors as more complex motor patterns emerge through maturation and experiential learning [59]. A delay in the reduction of MS in ASD may occur due to motor problems related to poor sensorimotor information integration [16, 59, 60]. Consequently, MS persistence is expected to be greater in younger individuals, with more severe developmental delays, poorer motor skills, higher ASD severity and greater deficits in sensory, emotional, cognitive, and motor integration [4, 9, 23, 24, 30, 44].

On the other hand, variations in the developmental trajectory of MS could be related to temporal changes in the context and in the motivators that elicit MS expression. MS increase in response to seeking sensory and proprioceptive stimuli in unstimulating environments [17, 54, 61, 62]. Conversely, MS decrease in predictable (less anxiety-inducing) and stimulus-rich settings [17, 63]. In stressful contexts, such as during activity transitions or social situations, MS can help maintain focus and reduce anxiety [64]. Adults with ASD who avoid performing MS in public due to social stigma may experience heightened anxiety and reduced capacity to manage sensory overload [27].

Regarding the comparison subgroups, no statistically significant differences were observed between male and female participants with ASD in terms of the frequency, severity, or types of MS per case. This finding aligns with the results of six out of seven studies reviewed by Melo *et al*. [9], who were unable to perform a meta-analysis because of insufficient data. However, the low percentage of women in our sample limits the conclusiveness of these results.

In the comparison analysis of ASD with and without ID, the ASD with ID group was found to exhibit more types of MS per case, consistent with previous studies [9, 26, 34, 44]. In 2009, Goldman *et al*. [24] suggested that the correlation of MS with the severity of autism and ID, both markers of underlying dopamine-mediated subcortical dysfunction, indicates a direct neurobiological origin of MS. This finding aligns with the consistent presence of MS in syndromic autism with ID, attributed to known genetic variants [65]. Adolescents and adults who have ASD without ID appear to use MS as a homeostatic mechanism to manage emotional dysregulation, alongside other more cognitively elaborate strategies, albeit in a less overt, more adaptive, and effective manner than their counterparts with ASD and ID. Conversely, in individuals with ASD and ID, the greater use of MS may stem from poorer integration of sensory, emotional, cognitive (intellectual and executive function), and motor information, leading to an imbalance that is not fully compensated for by these possibly maladaptive coping strategies [4, 62]. While significant differences in frequency and severity between ASD with and without ID might have been expected, such differences may not have been detected because of the study’s use of a simple IQ cut-off point of 70 to separate groups rather than accounting for the severity of ID. Additionally, only 35.6% of cases in our sample had ID, and MS was also prevalent in ASD cases without ID (84%), resulting in minimal differences. This observation is consistent with findings by Leekam *et al*. [4] in 2011, which highlighted the persistence of low-level RRBs in adults with ASD who have good cognitive ability. Differences compared with the studies in the review by Melo *et al*. [9] may also be attributed to the use of different scales to quantify complex repetitive MS, which vary in their measures of frequency, severity, or periodicity [27, 66].

The differences observed in the prevalence, frequency, severity, and types of MS per case between groups with and without psychopathological comorbidities could be explained by the use of MS by individuals with ASD as mechanisms for coping, emotional regulation, anxiety reduction [46, 55], or uncertainty reduction [16, 33, 67]. Numerous studies have demonstrated an association between anxiety and MS in ASD [15, 31, 32, 33, 68]. Additionally, ASD has a high rate of psychiatric comorbidity (up to 70%) [69], which was also observed in our study, with a psychiatric history of 59.6%. Moreover, MS is also present in other psychiatric disorders, and the coexistence of psychopathological comorbidities could add further risk for MS [4]. MS has been reported to affect functioning and social adjustment [70], which may secondarily lead to clinically significant distress. A comprehensive review of psychiatric comorbidities and their influence on the occurrence of MS is essential, particularly because these comorbidities may be treatable.

This study has several limitations. The sample size may have influenced the statistical significance of our findings. The sample size is relatively small for the number of MS studied. The low percentage of female participants and cases with ID limits the ability to draw conclusive results; however, these proportions reflect the prevalence of such cases in the clinical population. Other factors that may influence MS, such as motor skills, ASD severity, emotional dysregulation, sensory reactivity, or executive function, have not been analyzed [9, 28, 29, 30, 31, 32, 33, 34, 35]. Retrospective data collection may have introduced recall bias, and the definition of past MS as those occurring more than 1 year ago could be considered controversial. Regarding the use of the SBS as a measure of MS in ASD, it should be noted that the scale was designed for adolescents and adults with ID and has not been validated specifically for the ASD population, although it has been used in such contexts in the literature [71, 72]. Nevertheless, we opted for this scale over others more commonly used in the literature [21, 73] because it provides a more comprehensive description of MS [37]. To focus exclusively on MS, we developed a modified version of the scale (SBS-MS), excluding sensory items and those referring to object use, following approaches used by other authors [74], but this version has not been validated specifically for ASD populations, which may affect the reliability of the results.

Regarding the use of ASEBA to measure comorbidity, there is an overlap between ASD symptoms and some of its subscales, which means that differences between groups with and without psychopathological comorbidities could potentially reflect the severity of ASD rather than true comorbidity differences [75, 76]. Despite this limitation, the ASEBA was suitable for use in our study because of its availability in versions for all ages, the option for self- or heteroadministration, and the ability to convert scores into qualitative variables, enabling pooled analysis. With respect to the use of self- or heteroadministered scales, caregiver-reported data was obtained only when participants were unable to understand or complete questionnaires. Mazefsky *et al*. [77] highlighted that children and adolescents with ASD typically have limited emotional awareness and insight. By contrast, Jiujias *et al*. [67] reported that adults with ASD align closely with their parents’ personality scores, suggesting that they possess relatively good insight. In addition, social stigma may cause MS to be hidden in adults with ASD cognitive able, therefore this behavior will be less prevalent in studies based on observations or parent’s reports [44, 64].

## Conclusion

MS persisted with high frequency into adolescence and adulthood but decreased in both frequency and severity. There was no significant influence of age or sex on the types, frequency, or severity of MS in this age group.

Certain types of MS, particularly those considered more socially inappropriate, were more likely to diminish over time. Hand movements and pacing were the most prevalent MS, maintaining their periodicity compared to the past. While repetitive body movements were also prevalent, their periodicity decreased over time.

Groups with ASD and ID, as well as those with ASD and psychopathological comorbidities, exhibited more types of MS per case than their comparison groups. MS were also more frequent and severe in individuals with ASD and psychopathological comorbidities.

Studying typology, frequency, severity, and evolution of MS, along with their associations with other variables and contexts, could aid in predicting the progression of MS. This understanding could contribute to the design of more specific treatments for MS (if needed) and the identification of phenotypic subgroups, thereby facilitating the discovery of associated risk genes.

## Availability of Data and Materials

The datasets used and/or analyzed during the current study are available at the corresponding author on reasonable request.
